# A Defect in Influenza A Virus Particle Assembly Specific to Primary Human Macrophages

**DOI:** 10.1128/mBio.01916-18

**Published:** 2018-10-23

**Authors:** Sukhmani Bedi, Takeshi Noda, Yoshihiro Kawaoka, Akira Ono

**Affiliations:** a Department of Microbiology and Immunology, University of Michigan Medical School, Ann Arbor, Michigan, USA; b Laboratory of Ultrastructural Virology, Department of Virus Research, Institute for Frontier Life and Medical Sciences, Kyoto University, Kyoto, Japan; c Division of Virology, Department of Microbiology and Immunology, Institute of Medical Science, University of Tokyo, Tokyo, Japan; d Department of Pathobiological Sciences, University of Wisconsin—Madison, Madison, Wisconsin, USA; NIAID, NIH

**Keywords:** actin, influenza, macrophages, plasma membrane, virus assembly

## Abstract

Influenza A virus (IAV) propagates efficiently in epithelial cells, its primary target in the respiratory tract. In contrast, productive infection of most IAV strains is either blocked or highly inefficient in macrophages. The exact nature of the defect in IAV replication in human macrophages remains unknown. In this study, we showed that even compared to a monocytic cell line differentiated to macrophage-like cells, primary human monocyte-derived macrophages (MDM) are inefficient in IAV production, despite comparable levels of expression of viral glycoproteins at the plasma membrane. Correlative fluorescence scanning electron microscopy revealed that formation of budding structures at the cell surface is inefficient in MDM even though clustering of a viral glycoprotein, hemagglutinin (HA), is observed, suggesting that a step in IAV particle assembly is blocked in MDM. Using an *in situ* proximity ligation assay, we further determined that HA associates with neuraminidase (NA) but fails to associate with another viral transmembrane protein, M2, at the MDM plasma membrane. Notably, the defects in HA-M2 association and particle assembly in MDM were reversed upon cytochalasin D treatment that inhibits actin polymerization. These results suggest that HA-M2 association on the plasma membrane is a discrete step in IAV production, which is susceptible to suppression by actin cytoskeleton in MDM. Virus release remained inefficient in MDM upon cytochalasin D treatment, suggesting the presence of an additional defect(s) in virus release in this cell type. Overall, our study revealed the presence of multiple cell-type-specific mechanisms negatively regulating IAV production at the plasma membrane in MDM.

## Abstract (precis)

Identification of host cell determinants promoting or suppressing replication of viruses has been aided by analyses of host cells that impose inherent blocks on viral replication. In this study, we show that primary human MDM, which are not permissive to IAV replication, fail to support virus particle formation. This defect is specific to primary human macrophages, since a human monocytic cell line differentiated to macrophage-like cells supports IAV particle formation. We further identified association between two viral transmembrane proteins, HA and M2, on the cell surface as a discrete assembly step, which is defective in MDM. Defective HA-M2 association and particle budding, but not virus release, in MDM are rescued by disruption of actin cytoskeleton, revealing a previously unknown, negative role for actin, which specifically targets an early step in the multistep IAV production. Overall, our study uncovered a host-mediated restriction of association between viral transmembrane components during IAV assembly.

## INTRODUCTION

Influenza A virus (IAV) is a negative-strand RNA virus that mainly infects and replicates in epithelial cells in the respiratory tract. However, the virus has also been shown to infect other cell types such as macrophages, dendritic cells, and mast cells *ex vivo* (1–3). Host-cell-specific differences have been observed for various properties of IAV, including morphology and replication (for example, see references 4 to 8). These differences could be due to differences in expression levels or functions of host cellular proteins between cell types. In cases where cell-type-specific differences affect productive infection of a virus, detailed comparison between permissive and nonpermissive cell types often leads to identification of virus cofactors (7, 9–12) or host factors that restrict replication of viruses (8, 13–16). This approach, which often determines the specific function of the host factor of interest even prior to the identity of the factor, can serve as a complementary approach to genome-wide approaches (17–26).

*Ex vivo* infection studies have shown that in comparison to epithelial cells, macrophages are less permissive or nonpermissive to productive infection of seasonal IAV strains (27–33). Murine macrophages are nonpermissive to IAV replication (27, 29, 33, 34). Primary human blood-derived or alveolar macrophages do support seasonal IAV replication at detectable levels, although they are still much less permissive to virus growth than human epithelial cells (28, 30, 31, 34). As for the defective stages of the IAV life cycle, a block at the entry stage of infection has been identified in murine macrophages for most H1N1 strains (27, 29, 33). In addition, the presence of a defect(s) at a later stage has been known for IAV infection in murine macrophages (29, 33). However, there are apparently conflicting data as to whether the defect is at pre- or posttranslation stage (29, 33). Moreover, the mechanism in either case has yet to be determined. In contrast to murine macrophages, human macrophages support early stages of replication of all tested IAV strains yet are unable to complete the virus life cycle (33). While the defect appears to be posttranslational, the exact nature of this defect in human macrophages and the molecular mechanism behind it are not known.

Determining the nature of the human macrophage-specific defect in IAV replication is likely to advance our understanding of the roles played by cellular functions in late phases of the IAV life cycle and potentially facilitate identification of human host factors involved in this process. In the current study, we used primary human monocyte-derived macrophages (MDM) in order to identify the defective step in IAV replication in human macrophages. We show that MDM support early stages of the IAV assembly process, i.e., trafficking of the viral glycoproteins hemagglutinin (HA), neuraminidase (NA), and the ion channel protein M2 to the plasma membrane, but are inefficient at virus particle formation and subsequent virus release. This defect in virus particle formation and release is specific to primary MDM, since a monocytic cell line, THP1, when differentiated into macrophage-like cells, supports efficient virus particle production. Notably, we observed that the association of HA with M2 on the plasma membrane, as determined by the close proximity of <40 nm, is highly inefficient in MDM relative to the differentiated THP1 cells. In contrast, HA and NA associate efficiently on the surface of MDM. The defective association between HA and M2 is rescued in MDM upon treatment with an actin polymerization inhibitor, cytochalasin D, whereas this defect is recreated in differentiated THP1 cells by treatment with jasplakinolide (Jasp), which promotes actin polymerization. Consistent with the restoration of HA-M2 association in MDM, treatment with cytochalasin D also increases formation of budding structures in this cell type. However, virus release is not restored in MDM upon cytochalasin D treatment, suggesting the presence of an additional block in IAV assembly/release in this cell type. Overall, this study has identified virus particle formation, more specifically association between HA and M2, as a step defective in the IAV life cycle in primary human macrophages and revealed that this macrophage-specific block of IAV assembly requires actin polymerization.

## RESULTS

### MDM are inefficient in supporting productive IAV infection relative to differentiated THP1 cells.

**FIG 1:**
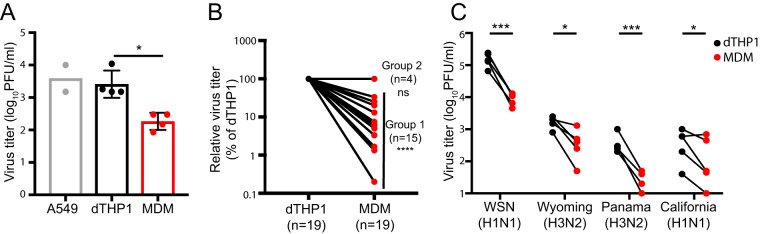
MDM are defective in productive IAV infection. (A) A549 cells, dTHP1 cells, and MDM were infected with WSN at MOI 0.01. Infectious virus titers in culture supernatants were measured at 11 hpi. (B) Infectious virus titers in culture supernatants were measured for WSN-infected dTHP1 cells and MDM at 24 hpi. For all tested donors, the relative virus titers in MDM cultures were calculated in comparison to the titer in dTHP1 cell cultures tested in parallel within the same experiment. Two groups of donors (groups 1 and 2) were designated based on the reduction in the titers or lack thereof. For group 1, the values for virus titers were in the range of 1.9 to 5.07 log_10_ PFU/ml. For group 2, the values were identical to those of corresponding dTHP1 cultures and in the range of 4.39 to 5.17 log_10_ PFU/ml. (C) dTHP1 and MDM (group 1) were infected with the given IAV strains at MOI 0.01, and infectious virus titers in culture supernatants at 24 hpi were determined by plaque assays using MDCK cells that have been passaged 20 to 30 times. Each circle represents an independently prepared culture. A black and a red circle connected by a line represent each independent experiment. For panel A, data are shown as mean ± SD. *, *P* < 0.05; ***, *P* < 0.001; ****, *P* < 0.0001; ns, nonsignificant.

To determine the extent to which human epithelial cells and macrophages differ in their ability to support productive IAV infection, we compared infectious IAV release from three different human cell types: the lung-derived epithelial cell line A549; the monocytic cell line THP1, which has been differentiated to adopt macrophage-like morphology (dTHP1); and primary monocyte-derived macrophages (MDM). The dTHP1 cells were obtained via treatment of THP1 cells with phorbol 12-myristate 13-acetate (PMA) and vitamin D3 for 2 to 3 days. A549, dTHP1, and MDM were infected with the laboratory strain A/WSN/1933 (H1N1) (WSN) at a multiplicity of infection (MOI) of 0.01 based on the PFU of virus stocks determined using MDCK cells. At 11 h postinfection (hpi), we observed that virus titers in MDM culture supernatants were up to 100-fold reduced in comparison to that in A549 culture supernatants. Unexpectedly, virus titers in culture supernatants were similar between A549 and dTHP1 cells (Fig. 1A). Since dTHP1 cells support influenza virus replication efficiently unlike MDM and yet belong to the same cellular lineage, to facilitate the analyses of the MDM-specific defect(s), we chose to compare IAV replication in MDM with that in dTHP1 cells in subsequent experiments. We noticed that while MDM isolated from the vast majority of the tested human donors showed a defect in productive IAV infection relative to dTHP1 at 24 hpi (denoted as group 1 in Fig. 1B), MDM from some donors (denoted as group 2 in Fig. 1B; ∼20%) showed no significant difference. Therefore, to identify the MDM-specific defect, the subsequent experiments were performed using MDM from the donors in group 1. In particular, in the mechanistic experiments (see Fig. 3 to 7), we verified in each experiment that MDM used show 10- to 20-fold reduction in the supernatant virus titers or released vRNA relative to dTHP1 cells at the indicated time point of the corresponding assays (data not shown).

To assess whether other IAV strains also replicate inefficiently in MDM relative to dTHP1 cells, we compared productive infection in dTHP1 cells and MDM of three previously or currently circulating IAV strains, in addition to WSN: A/Wyoming/03/2003 (H3N2) [Wyoming (H3N2)], A/Panama/2007/1999 (H3N2) [Panama (H3N2)], and A/California/04/2009 (H1N1) [California (H1N1)]. Infectious virus titers of all tested IAV strains, as measured by the plaque assay, were reduced by 10- to 50-fold in MDM in comparison to dTHP1 cells (Fig. 1C). These data suggest that MDM are highly inefficient at producing infectious IAV particles in comparison to dTHP1 cells.

### Both efficiency of virus release and infectivity of released particles are impaired in infected MDM relative to infected dTHP1 cells.

**FIG 2:**
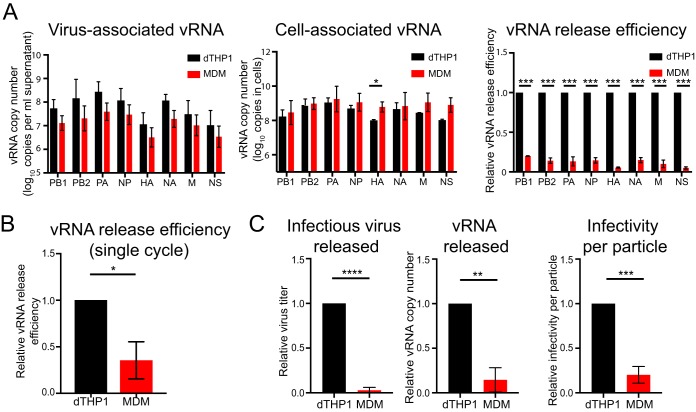
Both efficiency of virus release and infectivity of released particles are lower in MDM than in dTHP1 cells. dTHP1 cells and MDM were infected with WSN at MOI 0.1. (A) vRNA copy numbers were measured in lysates of virus pelleted from cell culture supernatants and cell lysates at 20 hpi. vRNA release efficiency was calculated as the ratio of number of vRNA copies in virus lysates to total number of vRNA copies (cell + virus) for each vRNA segment. Note that in most cases (except for cell-associated HA vRNA) differences between dTHP1 cells and MDM were not significant for virus-associated and cell-associated vRNA levels, which were compared as pooled data. However, vRNA release efficiency calculated for individual experiments showed a significant reduction in MDM cultures relative to dTHP1 cell cultures. (B) vRNA release efficiency was measured for infected dTHP1 cells and MDM at 12 hpi. To prevent the second cycle of infection, 10 μg/ml C179 was added to the cultures at 2 hpi. (C) PB2 vRNA copy number and virus titer were measured in dTHP1 and MDM culture supernatants at 16 hpi. Infectivity per particle was calculated as the ratio of virus titer to PB2 vRNA copy number in culture supernatants. Data are from experiments done with MDM from at least three independent donors and shown as mean ± SD. *, *P* < 0.05; **, *P* < 0.01; ***, *P* < 0.001, ****, *P* < 0.0001.

The results shown above and the results of time course experiments suggest that infectious virus release is reduced in MDM relative to dTHP1 cells even though flow cytometry using anti-vRNP antibody (clone 61A5 [35]) showed that similar fractions of cells in the cultures are infected (Fig. 1; see also Fig. S1 in the supplemental material). We sought to address whether the reduction in viral titers in MDM culture supernatants is due to a reduction in infectivity of released particles or whether it is due to a reduction in release of physical particles. To this end, we used viral RNA (vRNA) release as a surrogate to measure release of physical particles from dTHP1 cells and MDM. Numbers of vRNA copies released from MDM were 7- to 8-fold reduced in comparison to that from dTHP1 cells for all eight vRNA segments. However, there were no significant decreases in cell-associated vRNA levels in MDM relative to dTHP1 cells, indicating that MDM support viral RNA replication and earlier steps as efficiently as dTHP1 cells. vRNA release efficiency was calculated as the ratio of number of vRNA copies in virus pellets from cell culture supernatants to the total number of vRNA copies (cell + virus). For all eight vRNA segments, we observed a 5- to 10-fold reduction in vRNA release efficiency in MDM relative to dTHP1 cells (Fig. 2A). We also measured vRNA release efficiency in dTHP1 cells and MDM after a single round of virus replication (Fig. 2B). To block virus entry after the first round on infection, cells were treated with medium containing 10 μg/ml C179, a neutralizing antibody that binds to the HA stem (36, 37), at 2 hpi. At 12 hpi, we observed a 2- to 3-fold reduction in vRNA release efficiency in MDM relative to dTHP1 cells. These data suggest that efficiency of physical viral particle release from MDM is reduced in comparison to that from dTHP1 cells in the context of both single and multiple rounds of virus replication.

10.1128/mBio.01916-18.2 FIG S1 Infectious virus release is reduced in MDM cultures relative to dTHP1 cells despite similar numbers of infected cells in the two cultures. dTHP1 cells and MDM were infected with given IAV strains at MOI 0.01. At the indicated time points postinfection, culture supernatants and cells were collected and assessed for virus titer (top panels) and % vRNP^+^ cells (lower panels), respectively. Data from at least three independent experiments are shown as mean ± SD. *, *P* < 0.05; **, *P* < 0.01. Download FIG S1, EPS file, 0.9 MB. Copyright © 2018 Bedi et al. 2018 Bedi et al. This content is distributed under the terms of the Creative Commons Attribution 4.0 International license.

Importantly, the reduction in vRNA release (7- to 8-fold) from MDM does not entirely account for reduction in infectious virus release (up to 50-fold) (Fig. 2C). The ratio of released PFU (representing infectious virions) to released vRNA (representing total number of particles) was calculated as the infectivity per particle. Infectivity per particle for virus particles released from MDM was 5- to 6-fold reduced versus that released from dTHP1 cells (Fig. 2C). Overall, our data suggest that the total number of virus particles released as well as the infectivity of released virus particles is reduced in MDM relative to dTHP1 cells.

### Formation of budding structures is inefficient in MDM relative to dTHP1 cells despite similar levels of viral glycoprotein expression at the plasma membrane.

**FIG 3:**
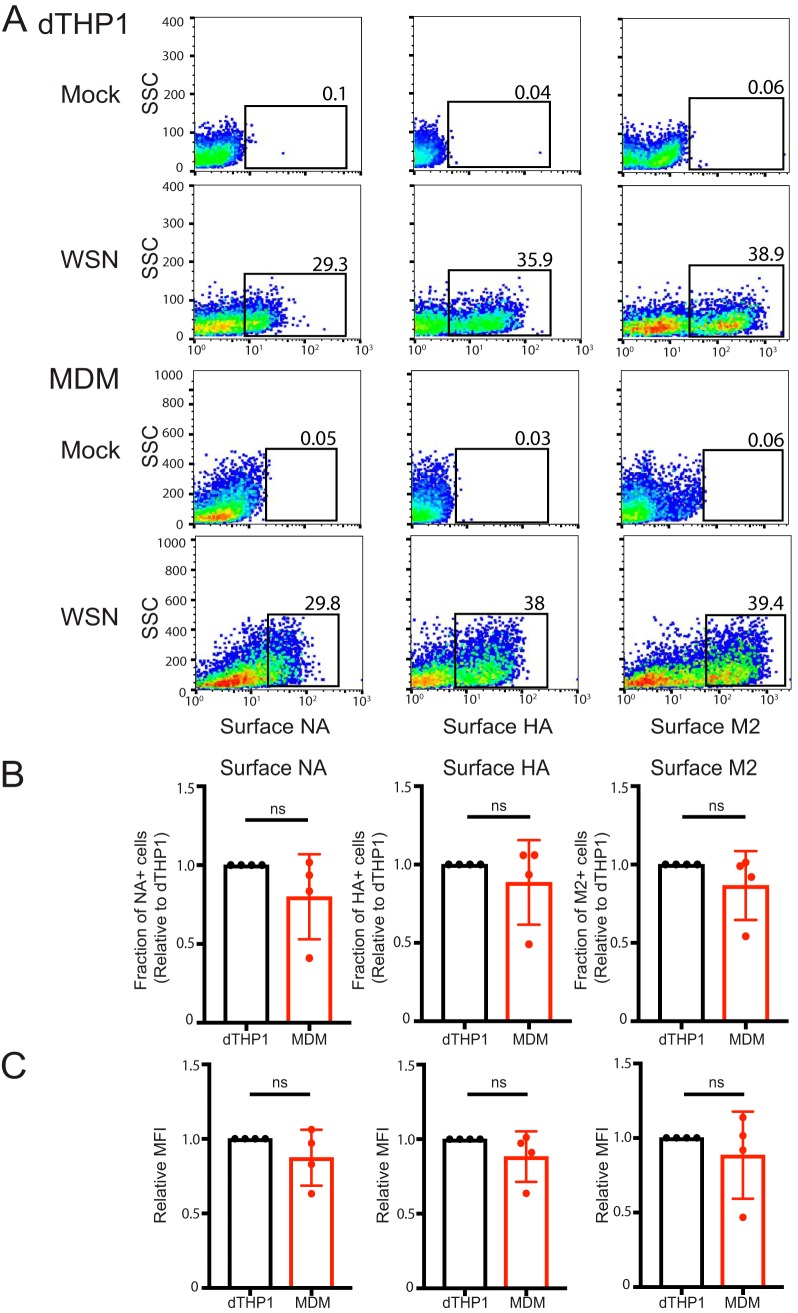
MDM are efficient at trafficking of viral glycoproteins to the cell surface. dTHP1 cells and MDM were infected with WSN at MOI 0.1. (A) Infected cells were analyzed for cell surface expression of HA, NA, and M2 by flow cytometry at 16 hpi. Representative flow plots for mock-infected (top row) and virus-infected (bottom row) cells are shown. Percentages of cells positive for viral proteins (boxed) are shown. Due to differences in the side scatter (SSC) profile between dTHP1 cells and MDM, the *y* axis (SSC) range is different between the two cell types. (B) Percentages of cells positive for surface expression of NA, HA, and M2 are compared between dTHP1 and MDM. (C) Relative MFIs for surface signal of indicated proteins for positive cell populations (gated in panel A) are shown. Data are shown as mean ± SD and are from at least three independent experiments. ns, nonsignificant.

We observed using flow cytometry that total expression levels of vRNP, HA, and M1 are comparable between dTHP1 cells and MDM in both the size of positive cell populations and the expression levels per cell (Fig. S1 and S2), indicating that protein translation and earlier steps are unlikely to be impaired in MDM. Henceforth, we focused on steps after viral protein translation: virus assembly, budding, and release. Virus assembly is initiated by targeting of the glycoproteins HA and NA to the plasma membrane (38–40). The third transmembrane protein M2 is also recruited to the assembly sites at the plasma membrane and allows for completion of the virus budding process (39, 41). To determine whether trafficking of the three glycoproteins occurs similarly in dTHP1 cells and MDM, we next compared levels of HA, NA, and M2 proteins on the surface of WSN-infected cells. We found that sizes of cell populations positive for surface expression of the three proteins are comparable between MDM and dTHP1 cells (Fig. 3A and B). The mean fluorescence intensity (MFI) for the three viral proteins in positive cell populations was also similar between MDM and dTHP1 cells (Fig. 3C), indicating that trafficking of viral glycoproteins to the plasma membrane is comparable between MDM and dTHP1 cells.

10.1128/mBio.01916-18.3 FIG S2 MDM express viral proteins as efficiently as dTHP1 cells. dTHP1 cells and MDM were infected with WSN at MOI 0.01 for 24 hours. Infected cells were fixed, permeabilized, and analyzed for cellular levels of vRNP, HA, and M1 by flow cytometry. (A) Representative flow plots for mock- and WSN-infected cells are shown. Gates for positive cell populations were set in comparison to mock-infected cells. Due to differences in the side scatter (SSC) profile between dTHP1 cells and MDM, the *y* axis (SSC) range is different between the two cell types. (B) Percentages of cells positive for vRNP, HA, and M1 are compared between dTHP1 and MDM. (C) MFIs (normalized to dTHP1 cells) for all three viral components are shown for positive cell populations (gated in panel A). Data are from at least three independent experiments and are shown as mean ± SD. ns, nonsignificant. Download FIG S2, EPS file, 2.2 MB. Copyright © 2018 Bedi et al. 2018 Bedi et al. This content is distributed under the terms of the Creative Commons Attribution 4.0 International license.

**FIG 4:**
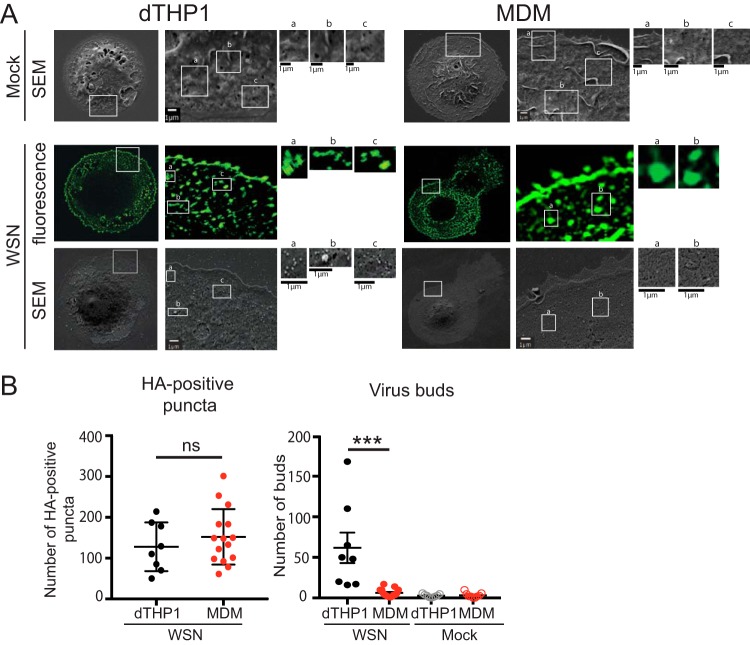
MDM are defective in virus bud formation despite expression of HA on the cell surface. dTHP1 cells and MDM grown on gridded coverslips were infected with WSN at MOI 0.1 for 20 h. Cells were fixed and immunostained with anti-HA. After identification of HA-positive cells by confocal fluorescence microscopy, cells were prepared for SEM. The same cells were identified based on grid positions and analyzed by SEM. (A) Representative SEM images for mock-infected and WSN-infected HA-positive cells are shown in the top and bottom rows, respectively. Fluorescence images corresponding to the SEM images of WSN-infected cells are shown in the middle row. Boxed areas are magnified and shown on the right of original images. Alphabetic labels are used to distinguish between individual boxed areas. (B) The numbers of HA-positive puncta identified in fluorescence images (left panel) and ∼100-nm buds identified in SEM images (right panel) were counted within the same-size area (100 μm^2^ in size) in each cell. Data are shown for 8 to 15 cells from two independent experiments. Error bars represent standard error of mean. ***, *P* < 0.0001; ns, nonsignificant.

We next asked whether dTHP1 cells and MDM expressing HA on the cell surface support virus particle formation. To address this question in a single cell basis, we performed correlative fluorescence and scanning electron microscopy (CFSEM) in which we first identify cells with surface HA expression using fluorescence microscopy and then examine formation of virus particle-like buds on the surface of the same cells using scanning electron microscopy (SEM). Fluorescence microscopy showed that HA is uniformly distributed on the surface of both dTHP1 cells and MDM with some local accumulation. These HA-enriched clusters or puncta, which likely represent sites of virus assembly, were clearly distinguished on the surface of infected cells after the median filter was applied to the confocal images to remove signal for uniformly distributed nonpunctate HA. These HA-enriched sites often corresponded to budding structures with a diameter of approximately 100 nm on the surface of WSN-infected dTHP1 cells (Fig. 4A). Very few budding structures with the similar size were observed on the surface of mock-infected cells. MDM also form ∼100-nm virus particle-like buds on the surface in HA-positive cells, albeit the number of buds observed in MDM was markedly lower than in dTHP1 cells (Fig. 4A). To assess the formation of budding structures quantitatively, we counted the number of HA-positive puncta and the number of virus particle-like buds within the same-sized area (100 μm^2^ in size) of each cell. Even though MDM showed higher numbers of HA-positive puncta on the cell surface than dTHP1 cells, the numbers of virus buds were drastically reduced in MDM relative to dTHP1 cells (Fig. 4B). Overall, these results indicate that virus particle assembly and budding are inefficient in MDM despite efficient trafficking of HA, NA, and M2 to the plasma membrane.

### Association between HA and M2 is impaired in MDM but not in dTHP1 cells.

**FIG 5:**
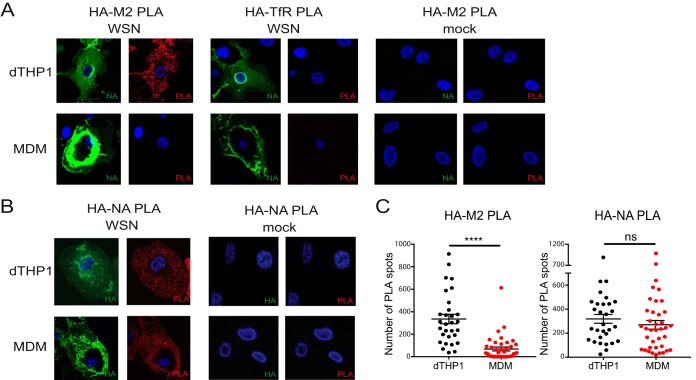
Association between HA and M2 is defective in MDM relative to dTHP1 cells. dTHP1 cells and MDM were infected with WSN at MOI 0.1 for 16 h. (A) Cells were examined by PLA using goat anti-HA and mouse anti-M2 or goat anti-HA and mouse anti-TfR antibodies. To identify infected cells, surface NA was also detected by rabbit anti-NA. Nuclei were stained with DAPI (blue). Representative maximum intensity projection images, which were reconstructed from Z-stacks corresponding to the focal planes ranging from the middle plane of the nucleus to the bottom of the cells, are shown. (B) Cells were examined by PLA using mouse anti-HA and rabbit anti-NA antibodies. Goat anti-HA was used for detection of infected cells. Representative maximum intensity projection images are shown as in panel A. Note regions of intense NA (in panel A) and HA (in panel B) signal on the surface of MDM due to the presence of membrane ruffles. (C) Number of PLA spots was counted for each cell. Data are shown for three independent experiments, and 8 to 10 cells were analyzed per experiment. These experiments were performed in parallel with the experiments shown in Fig. 3 using MDM from the same donors. Error bars represent standard error of mean. ****, *P* < 0.0001; ns, nonsignificant.

Based on results shown above, we hypothesize that local coenrichment of HA, NA, and M2, which leads to formation of virus assembly sites, is not efficient in MDM relative to dTHP1 cells. To compare formation of the putative assembly sites between dTHP1 cells and MDM, we used *in situ* proximity ligation assay (PLA). PLA allows for detection of two proteins localized within 40-nm distance of each other and has been used to visualize IAV assembly sites on the plasma membrane (42). In addition to measuring PLA signal between the given pair of proteins, we also costained cells for cell surface NA to identify infected cells. As a negative control, we performed PLA between HA and transferrin receptor (TfR). TfR does not associate with lipid rafts (43), the plasma membrane microdomains associated with IAV assembly sites (39, 44). Infected dTHP1 cells showed high PLA signal for HA-M2 association. In contrast, infected MDM showed very few PLA spots between HA and M2 (Fig. 5A and C). As expected, no PLA signal was observed between HA and M2 in mock-infected cells or between HA and TfR in infected cells (Fig. 5A). The majority (80% to 90%) of surface NA-positive MDM and dTHP1 cells express HA and M2 on their surface at comparable levels (Fig. S3). Therefore, the significant reduction in HA-M2 PLA signal in MDM relative to dTHP1 cells is not due to the lack of expression of HA and/or M2 in NA-positive cells.

10.1128/mBio.01916-18.4 FIG S3 Majority of NA-expressing dTHP1 cells and MDM coexpress both HA and M2. dTHP1 cells and MDM were infected with WSN at MOI 0.1 for 16 hours. Cells were fixed and stained for surface HA, M2, and NA. Representative plots are shown in the left panel. % cells expressing HA and M2 within the NA-positive cell population were determined and shown in the right panel. Data are from at least three independent experiments and shown as mean ± SD. ns, nonsignificant. Download FIG S3, EPS file, 1.0 MB. Copyright © 2018 Bedi et al. 2018 Bedi et al. This content is distributed under the terms of the Creative Commons Attribution 4.0 International license.

To determine whether the defect in association between transmembrane proteins in MDM is specific to HA and M2 or whether association between other pairs of viral transmembrane proteins is defective as well, we next measured PLA signal between HA and NA. In this case, to identify infected cells, we costained cells for cell surface HA using an antibody different from the one used for PLA. PLA signal between HA and NA was similar for dTHP1 cells and MDM, suggesting that HA and NA associate with each other as efficiently on the surface of MDM as on dTHP1 cells (Fig. 5B and C). Overall, our data indicate that association between HA and M2 is a virus assembly step specifically impaired in MDM.

### Inhibition of actin polymerization increases HA-M2 PLA and bud formation in MDM.

**FIG 6:**
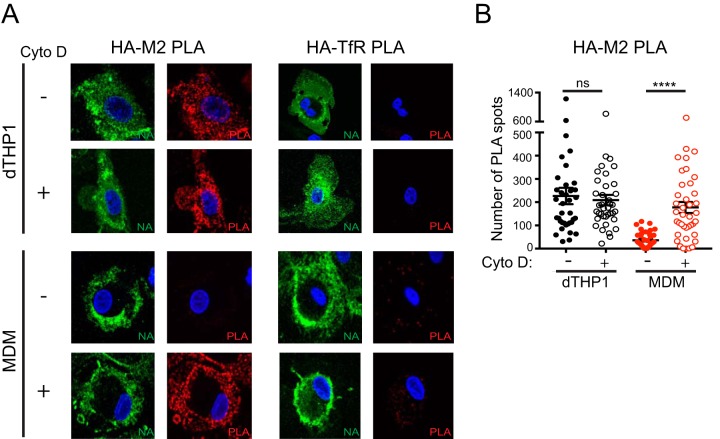
Cytochalasin D treatment restores HA-M2 PLA in MDM to levels comparable to that in dTHP1 cells. dTHP1 cells and MDM were infected with WSN at MOI 0.1. At 14 hpi, cells were treated with vehicle control (DMSO) or 20 μM cytochalasin D (Cyto D) for 2 h before fixation. (A) Cells were analyzed as in Fig. 5A. Representative maximum intensity projection images are shown. (B) Number of PLA spots was counted for each cell. These experiments were performed in parallel with the experiments shown in Fig. S4 using MDM from the same donors. Data are from at least three independent experiments, and 8 to 10 cells were analyzed per experiment. Error bars represent standard error of mean. ****, *P* < 0.0001; ns, nonsignificant.

HA associates with lipid rafts on the plasma membrane, while M2 mainly localizes in non-lipid raft areas (39, 44). It is suggested that M2 is recruited to cholesterol-rich lipid rafts during IAV particle assembly (45, 46); however, host cell functions and factors that regulate this step are not known. It is possible that in MDM, HA-containing plasma membrane microdomains stay segregated from those containing M2, leading to defective association between the two glycoproteins. The cortical actin cytoskeleton, a network of filaments that underlies and interacts with the plasma membrane, is suggested to play a role in formation and maintenance of plasma membrane microdomains (47–49). Therefore, we next asked whether the actin network regulates the association between HA and M2 in dTHP1 cells and MDM. Infected cells were treated with cytochalasin D (Cyto D), an inhibitor of actin polymerization, at 14 hpi for 2 h, fixed, and examined for HA-M2 association using PLA. Phalloidin staining confirmed that Cyto D disrupts the cellular actin network in both dTHP1 cells and MDM under these treatment conditions (Fig. S4A). Infected dTHP1 cells showed high PLA signal for HA-M2 association under both vehicle- and Cyto D-treated conditions. As observed in Fig. 5A, vehicle-treated MDM showed very few PLA spots between HA and M2. In contrast, Cyto D-treated MDM showed PLA signal between HA and M2 at levels similar to that observed for dTHP1 cells (Fig. 6A and B). No PLA signal was observed between HA and TfR in untreated or Cyto D-treated dTHP1 cells and MDM. The increase in HA-M2 PLA signal upon Cyto D treatment of MDM was not due to an increase in surface expression of HA and M2 in drug-treated cells, as shown by the flow cytometry analysis (Fig. S4B and C). These results suggest that actin polymerization suppresses HA-M2 association in MDM.

10.1128/mBio.01916-18.5 FIG S4 Effects of cytochalasin D treatment on the actin cytoskeleton, cell surface expression of viral transmembrane proteins, and released virus titers in dTHP1 cells and MDM. dTHP1 cells and MDM were infected with WSN at MOI 0.1 for 14 hours. Cells were treated with vehicle control (DMSO) or 20 μM Cyto D for 2 hours (A to C) or 4 hours (D). (A) Cells were fixed at 16 hpi, and the actin cytoskeleton was visualized using fluorescently tagged phalloidin. Images are representative of three independent experiments with 10 cells visualized per experiment. An image with enhanced brightness is also shown for Cyto D-treated MDM. (B and C) Cells were fixed at 16 hpi. % cells expressing HA, NA, and M2 on the cell surface (B) and MFIs for the indicated proteins in positive cell populations (C) are shown. (D) Infectious virus titers released in culture supernatants were measured at 18 hpi. Data are from three independent experiments and shown as mean ± SD. *, *P* < 0.05; **, *P* < 0.01; ***, *P* < 0.005. Download FIG S4, EPS file, 3.2 MB. Copyright © 2018 Bedi et al. 2018 Bedi et al. This content is distributed under the terms of the Creative Commons Attribution 4.0 International license.

**FIG 7:**
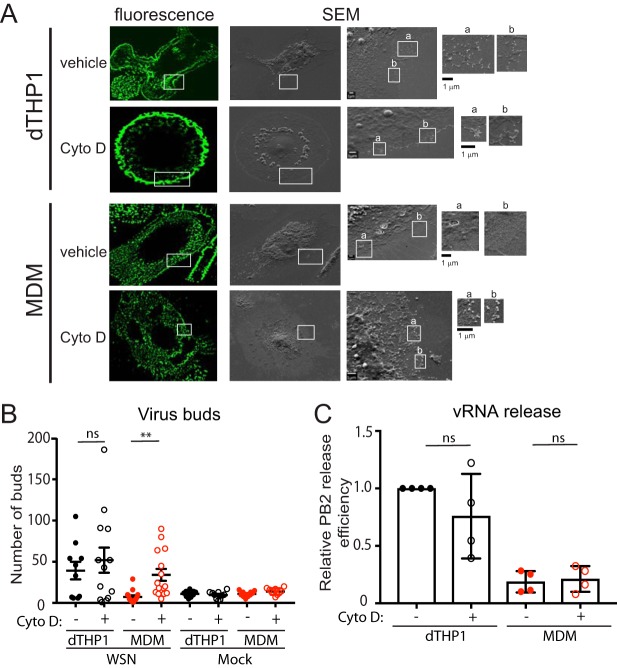
Cytochalasin D treatment increases bud formation in MDM to levels comparable to that in dTHP1 cells. dTHP1 cells and MDM were infected with WSN at MOI 0.1. At 14 hpi, cells were treated with vehicle control (DMSO) or 20 μM Cyto D for 4 h before fixation and immunostaining with anti-HA. After identification of HA-positive cells by confocal fluorescence microscopy, cells were processed for SEM. The same cells were identified based on grid positions and analyzed by SEM. (A) Representative SEM images for WSN-infected HA-positive cells are shown. Fluorescence images corresponding to the SEM images are also included. Boxed areas for SEM images are magnified and shown on the right of original images. Alphabetic labels are used to distinguish between the individual boxed areas. (B) The number of ∼100-nm buds identified in SEM images was counted within the same-size area (100 μm^2^ in size) in each cell. Data are shown for 10 to 20 cells from three independent experiments. (C) vRNA release efficiency was measured in infected MDM and dTHP1 cell cultures treated with DMSO or Cyto D for 4 h. For panel B, error bars represent standard error of mean. For panel C, error bars represent SD. **, *P* < 0.01; ns, nonsignificant.

We next asked whether treatment with Cyto D restores virus budding in MDM. To this end, we performed CFSEM of dTHP1 cells and MDM treated with either vehicle or Cyto D at 14 hpi for 4 h and examined virus bud formation in cells expressing HA on the cell surface. We counted the number of virus particle-like buds (∼100 nm in diameter) within the same-sized area (100 μm^2^ in size) of each cell. Consistent with the results shown in Fig. 4, vehicle-treated, HA-positive MDM showed significantly lower numbers of buds on the cell surface than vehicle-treated, HA-positive dTHP1 cells. Notably, Cyto D treatment significantly increased the number of buds on the surface of MDM to levels comparable to those in vehicle-treated dTHP1 cells (Fig. 7A and B). Cyto D-treated dTHP1 cells showed no significant increase in bud formation relative to vehicle-treated dTHP1 cells. Very few such budding structures corresponding to the size of IAV particles were observed on the surface of untreated or Cyto D-treated, mock-infected dTHP1 cells and MDM (Fig. 7B). These data suggest that disruption of the actin cytoskeleton promotes IAV particle assembly in MDM. To determine whether the increase in efficiency of bud formation leads to an increase in virus release, we measured the PB2 vRNA release efficiency as shown in Fig. 2A. Again, vRNA release efficiency was 4- to 5-fold reduced in vehicle-treated MDM cultures relative to vehicle-treated dTHP1 cell cultures. Contrary to expectation, Cyto D treatment did not enhance vRNA release efficiency (Fig. 7C). We also did not observe any increase in infectious virus titers (Fig. S4D) in supernatants of MDM cultures upon Cyto D treatment. These results indicate that disruption of the actin cytoskeleton promotes virus budding but not virus release. It is conceivable that there is an additional MDM-specific block in the late assembly/release stages of the IAV life cycle, which cannot be reversed by Cyto D. Overall, these data show that virus particle assembly, more specifically HA-M2 association, is negatively regulated by the actin cytoskeleton in MDM.

### Promotion of actin polymerization reduces HA-M2 PLA in dTHP1 cells.

**FIG 8:**
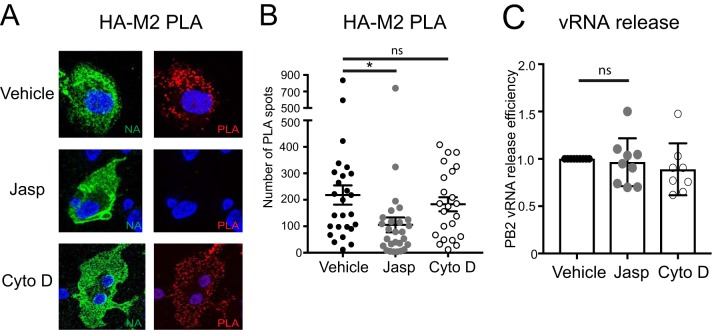
Jasplakinolide treatment reduces HA-M2 PLA in dTHP1 cells. dTHP1 cells were infected with WSN at MOI 0.1. (A and B) At 10 hpi, cells were treated with vehicle control (0.5% ethanol), 1 μM jasplakinolide (Jasp), or 20 μM Cyto D for 4 h before fixation. (A) Representative maximum intensity projection images are shown as in Fig. 5A. (B) Number of PLA spots was counted for each cell, and data are shown for 7 to 10 cells per experiment. (C) vRNA release efficiency was measured in infected dTHP1 cell cultures treated with vehicle, Jasp, and Cyto D for 4 h. Data are from at least three independent experiments. For panel B, error bars represent standard error of mean. For panel C, error bars represent SD. *, *P* < 0.05; ns, nonsignificant.

Since inhibition of actin polymerization restores association of HA and M2 in MDM, we next asked whether promoting actin polymerization inhibits HA-M2 association in dTHP1 cells. To this end, infected dTHP1 cells were treated with jasplakinolide (Jasp), which nucleates and stabilizes actin polymerization, at 14 hpi for 2 h and examined for HA-M2 association using PLA at 16 hpi as in Fig. 6. Two hours of Jasp treatment reduced HA-M2 PLA in 50% of the examined dTHP1 cells, while the remaining infected cell population showed HA-M2 PLA signal comparable to that in untreated cells (data not shown). We reasoned that high HA-M2 PLA signal in 50% of Jasp-treated dTHP1 cells is due to preexisting association between HA and M2 at the time of Jasp addition. Therefore, we next examined the effect of Jasp on HA-M2 association at an earlier time point in infection when preexisting HA-M2 coclusters are unlikely to be abundant. We treated infected dTHP1 cells with Jasp or Cyto D at 10 hpi for 4 h and examined for HA-M2 association using PLA at 14 hpi. Since Jasp and phalloidin compete for binding to the same site on F-actin (50), we were unable to use phalloidin staining to determine the effect of Jasp treatment on the actin cytoskeleton in dTHP1 cells. However, using an actin fractionation assay (51), we confirmed that treatment with Jasp for 4 h increases the ratio of insoluble (i.e., polymerized) actin to soluble actin in dTHP1 cells, in comparison to vehicle-treated cells (Fig. S5A). Under these conditions, most Jasp-treated dTHP1 cells showed reduced HA-M2 PLA signal, in comparison to vehicle- or Cyto D-treated cells (Fig. 8A and B). We note that surface HA and M2 expression in Jasp-treated cells was somewhat reduced relative to that in vehicle-treated cells (Fig. S5B and C). However, this reduction in surface expression of HA and M2 does not explain the decrease in HA-M2 PLA signal upon Jasp treatment; when HA or M2 expression on the cell surface and number of HA-M2 PLA spots were simultaneously assessed in the same cells, little correlation was observed between them (Fig. S6). Despite diminished HA-M2 association, vRNA release efficiency of Jasp-treated cells was not reduced in comparison to those of vehicle- and Cyto D-treated cells (Fig. 8C), suggesting that HA-M2 association, as measured by PLA, may not play as important a role in virus particle assembly/release in dTHP1 cells as it does in MDM (see Discussion). Consistent with this possibility, at 14 hpi, no obvious difference was observed in virus bud formation on the cell surface of dTHP1 cells following 4 h of treatment with Jasp versus vehicle or Cyto D (Fig. S7).

10.1128/mBio.01916-18.6 FIG S5 Effect of jasplakinolide treatment on the actin cytoskeleton and cell surface expression of HA and M2 in dTHP1 cells. (A) dTHP1 cells were treated with vehicle control (0.5% ethanol) or 1 μM Jasp for 4 hours. Cells were extracted with actin stabilization buffer and centrifuged. Pellet (P) and supernatant (S) fractions were analyzed using immunoblotting. (B, C) dTHP1 cells were infected with WSN at MOI 0.1 for 10 hours. Cells were treated with vehicle control or 1 μM Jasp for 4 hours. (B) % cells expressing HA and M2 on the cell surface are shown. (C) MFIs for the indicated proteins in positive cell populations are shown. Data are from three independent experiments and shown as mean ± SD. ns, nonsignificant; *, *P* < 0.05. Download FIG S5, EPS file, 6.3 MB. Copyright © 2018 Bedi et al. 2018 Bedi et al. This content is distributed under the terms of the Creative Commons Attribution 4.0 International license.

10.1128/mBio.01916-18.7 FIG S6 Weak correlation observed between the level of expression of HA or M2 and number of HA-M2 PLA spots in dTHP1 cells. dTHP1 cells were infected with WSN at MOI 0.1 for 10 hours. Cells were treated with vehicle control or 1 μM Jasp for 4 hours. (A) Cells were fixed and labeled with goat anti-HA and mouse anti-M2. Subsequently, cells were treated with PLA probes and then with fluorescently tagged secondary antibodies. Cells with similar levels of cell surface HA and M2 expression were compared for PLA signals. (B) Scatterplots between fluorescence intensities (FI) for cell surface HA or M2 signals and number of HA-M2 PLA spots are shown for vehicle- and Jasp-treated cells. The best-fit line was determined for each set of *x-y* data points using linear regression analyses. Correlation between the FI and PLA values was calculated as *R*^2^ values for each plot. Note that the correlations between FI and PLA values are generally low and that vehicle-treated cultures generally show higher PLA signals than Jasp-treated cultures compared at similar FI values. Data are pooled from two independent experiments. Download FIG S6, EPS file, 4.5 MB. Copyright © 2018 Bedi et al. 2018 Bedi et al. This content is distributed under the terms of the Creative Commons Attribution 4.0 International license.

10.1128/mBio.01916-18.8 FIG S7 Jasplakinolide treatment has no effect on virus bud formation in dTHP1 cells. dTHP1 cells were infected with WSN at MOI 0.1. At 10 hpi, cells were treated with vehicle control (0.5% ethanol), 1 μM Jasp, or 20 μM Cyto D for 4 hours before fixation and immunostaining with anti-HA. After identification of HA-positive cells by confocal fluorescence microscopy, cells were processed for SEM. The same cells were identified based on grid positions and analyzed by SEM. (A) Fluorescence and SEM images for representative WSN-infected HA-positive cells are shown. Boxed areas for SEM images are magnified and shown on the right of original images. Alphabetic labels are used to distinguish between individual boxed areas. (B) The number of ∼100-nm buds identified in SEM images was counted within the same-size area (100 μm^2^ in size) in each cell. Data are shown for 10 to 20 cells from two independent experiments. Error bars represent standard error of mean. Download FIG S7, EPS file, 39.9 MB. Copyright © 2018 Bedi et al. 2018 Bedi et al. This content is distributed under the terms of the Creative Commons Attribution 4.0 International license.

It is important to emphasize, however, that the defect in HA-M2 association is rescued upon inhibition of actin polymerization in MDM, while it is induced upon stabilization of actin in dTHP1 cells. Therefore, regardless of the effect on release of assembled particles, our data overall highlight a macrophage-specific role for actin polymerization in suppressing association between HA and M2 at the plasma membrane.

## DISCUSSION

In a previous study, a posttranslational defect in productive IAV infection was observed in human MDM (33). A similar defect was also reported for murine macrophages in one study (29) but not the other (33). The exact nature of these defects that lead to inefficient IAV production has not been determined. Here, we have shown that despite efficient trafficking of the viral glycoproteins to the cell surface (Fig. 3), infectious virus particle formation at the plasma membrane is inefficient in human MDM (Fig. 2 and 4). The current study further identified HA-M2 association as an IAV assembly step suppressed in MDM (Fig. 5). This restriction is specific to primary macrophages, as the THP1 monocytic cell line differentiated to macrophage-like cells (dTHP1 cells) supports HA-M2 association and efficient IAV production. Notably, defective HA-M2 association and bud formation in MDM can be ameliorated by the disruption of actin polymerization, revealing a role for the actin cytoskeleton in suppressing IAV particle assembly in MDM (Fig. 6 and 7). However, virus particle release remains inefficient even when HA-M2 association and bud formation are restored by actin disruption (Fig. 7C), implying the presence of an additional defect in a postassembly step in MDM. Consistent with the restrictive role of actin polymerization, HA-M2 association in dTHP1 cells was blocked upon a treatment that promotes actin polymerization (Fig. 8).

Previous studies observed strain-specific differences for IAV replication in human macrophages. Some strains such as highly pathogenic H5N1 and pandemic 1918 strains can replicate in macrophages albeit at a lower efficiency than in epithelial cells (27, 28, 31, 34). Marvin et al. recently reported that the laboratory strain WSN is able to overcome blocks in IAV replication in human macrophages, while replication of the A/California/04/2009 strain is completely blocked (33). In our study, we observed that all tested strains, including WSN and A/California/04/2009, released significantly lower titers in MDM than in dTHP1 cells (Fig. 1C). Thus, it is likely that the cell-type-specific difference observed in this study is distinct from the previously reported strain-specific difference.

In addition to identifying a defective step for IAV replication in primary human macrophages, our study also lends mechanistic insights into the assembly and budding process of IAV in host cells. IAV is thought to assemble in cholesterol-enriched microdomains, or membrane rafts, of the plasma membrane in host cells (52–55). HA and NA accumulate at these assembly sites (38–40, 44, 56), also known as the budozones, while the third transmembrane protein, M2, is suggested to localize at the edge of the budozone (41, 46, 57). Coclustering between HA and M2 has been observed at steady state in epithelial cells (45, 57, 58). However, the sequence of events leading to recruitment of M2 to the budozone is unknown. Whether there is a mechanism regulating these events, other than simple diffusion over the plasma membrane, also remains to be determined. Our PLA data suggest that different molecular mechanisms mediate association between HA and NA and association between HA and M2 at the plasma membrane. Consistent with this possibility, a recent study showed that NA but not M2 accelerates HA trafficking to the apical surface of epithelial cells, presumably through cotrafficking (56). Thus, recruitment of M2 to assembly sites enriched in HA (and perhaps NA) is a discrete and host-cell-dependent step in the IAV assembly process.

The actin cytoskeleton has been implicated in assembly of IAV particles, in particular formation of filamentous particles (6, 59, 60). However, the actin-dependent mechanism(s) regulating IAV assembly is not well understood. Previous studies have shown that disruption of actin dynamics by both inhibition of actin polymerization (6, 59) (by latrunculin A or cytochalasin D) and promotion of actin polymerization (59) (by jasplakinolide) disrupts filamentous IAV assembly. In contrast, our study showed that drugs inhibiting actin polymerization and depolymerization have distinct and opposing effects on HA-M2 association: blocking actin polymerization in IAV nonpermissive cells (MDM) restores HA-M2 association, whereas enhancement of polymerization in permissive cells (dTHP1 cells) reduces HA-M2 association. Therefore, it is likely that distinct actin-dependent mechanisms regulate the association between HA and M2 at the plasma membrane and formation of filamentous particles. As for the mechanism regulating HA-M2 association, one can speculate that subcortical actin promotes the segregation of HA- and M2-enriched plasma membrane microdomains. Consistent with this possibility, previous studies support a role for actin polymerization in maintaining HA-enriched microdomains as compact and dense (61, 62).

Of note, even though particle assembly is enhanced in MDM upon disruption of the actin cytoskeleton, virus release still remains defective in this cell type (Fig. 7). This suggests that an additional defect(s), for example, incomplete scission between viral envelope and plasma membrane or tethering of nascent particles to the cell surface, occurs in MDM. In addition to the nature of this late defect, whether the defect manifests due to the absence of a host factor enabling virus release or whether it is caused by the presence of a restriction factor blocking release of assembled virus particles warrants further investigation. It is also possible that cytochalasin D treatment blocks the release of assembled virus particles in an MDM-specific manner. These results, together with the pleiotropic effects of actin perturbation discussed above, also highlight the importance of specifically examining individual steps (e.g., HA-M2 association) rather than just monitoring the final outcomes, i.e., virus release efficiencies or released virus titers, when assessing the effect(s) of actin disruption on the virus assembly/release process.

The cytoplasmic domain of M2 contains an amphipathic helix that plays a role in scission of the IAV particle after budding (41, 57, 63). In epithelial (MDCK) or epithelial-like (293T) cell lines, viruses or VLP systems lacking M2 or expressing mutant M2 proteins are still able to initiate particle assembly and budding, presumably driven by HA and NA (38, 64). However, these buds adopt an abnormal morphology and/or fail to undergo scission or release (41, 57, 63), the latter of which results in accumulation of particles at the cell surface. In contrast, MDM showed very few buds on their surface under conditions where HA-M2 association was impaired (Fig. 4 and 7). These results suggest that in MDM, M2 plays an earlier role(s) in the assembly of virus particles, which is not apparent with 293T or MDCK cells that express M2-deficient virus or VLP systems. Such an earlier role may require other functions of M2. For example, in addition to scission of nascent particles, M2 functions in recruitment of M1 and vRNP to assembly sites (45, 63, 65–69), which is important for initiation of IAV particle assembly or elongation of filamentous particles (63, 68, 70–76). A defect in incorporation of M1 and/or vRNP into budding virus particles due to the failure of M2 recruitment may also explain the reduction in infectivity per particle observed for MDM-derived virus relative to dTHP1-derived virus (Fig. 2B). We also do not rule out the possibility that a failure in association of M1 and/or vRNP with the assembly sites may contribute to the observed defect in M2-HA association specific to MDM.

While jasplakinolide reduces HA-M2 association in dTHP1 cells, no reduction in virus bud formation is observed under this condition (see Fig. S7 in the supplemental material), which is consistent with previous studies performed with M2-lacking viruses in epithelial cell lines (41, 57, 63). However, we do not observe an arrest in virus release from dTHP1 cells, which was observed in the previous studies using M2-deficient viruses. In this regard, it is important to note that, unlike cells infected with M2-lacking mutant viruses, dTHP1 cells treated with jasplakinolide still show a residual level of M2 recruitment based on HA-M2 association detected by PLA. It is possible that this residual level of HA-M2 association is sufficient to promote the scission of virus buds in dTHP1 cells.

Overall, in this study, we have compared IAV replication in MDM with that in dTHP1 cells and found that MDM replicate vRNA, express viral proteins, and traffic HA, NA, and M2 to the plasma membrane at levels similar to those in dTHP1 cells. However, MDM are defective in assembling virus particles, likely due to actin-dependent suppression of association between the viral transmembrane proteins HA and M2. Comparison of actin regulatory mechanisms operating in MDM and dTHP1 cells, which are of the same lineage, will likely facilitate identification of additional host cellular factors involved in the assembly stage of the IAV life cycle.

## MATERIALS AND METHODS

### Cells and reagents.

Monocytes were isolated by plate adhesion from peripheral blood mononuclear cells, which were obtained from buffy coats derived from unidentified healthy donors (New York Blood Center, NY). Cells were cultured in RPMI 1640 (Gibco) supplemented with 10% fetal bovine serum (FBS, HyClone) for 7 days before they were used for experiments. THP1 (ATCC TIB202) cells were cultured in RPMI 1640 supplemented with 10% FBS, 1 mM sodium pyruvate (Gibco), and 0.05 mM 2­mercaptoethanol. To generate differentiated THP1 cells (dTHP1), THP1 cells were cultured in the medium containing 0.1 μM phorbol 12-myristate 13-acetate (PMA; Sigma) and 0.1 μM vitamin D3 (Sigma) for 2 to 3 days. Madin-Darby canine kidney (MDCK) cells were provided by Arnold S. Monto (University of Michigan) and were cultured in DMEM (Gibco) supplemented with 10% FBS and 25 mM HEPES. Human lung carcinoma cell line A549 was provided by Mike Bachman (University of Michigan) and was cultured in DMEM (Gibco) supplemented with 10% FBS and 25 mM HEPES. The human embryonic kidney-derived 293T cell line (ATCC) was cultured and maintained in DMEM (Lonza) supplemented with 10% FBS.

The following antibodies were used for immunofluorescence microscopy: mouse anti-HA monoclonal antibody (clone C179 [36]; TaKaRa), mouse anti-M2 monoclonal antibody (clone 14C2 [77]; ThermoFisher), mouse anti-vRNP monoclonal antibody (clone 61A5 [35]; a kind gift from Fumitaka Momose, Kitasato University), goat anti-HA antiserum (BEI NR-3148), mouse anti-transferrin receptor (TfR) monoclonal antibody (clone M-A712; BD Biosciences). Rabbit anti-NA antiserum was a kind gift from Christopher Brooke (University of Illinois). Mouse anti-actin (ACTN05) was purchased from ThermoFisher. All secondary antibodies used for immunofluorescence and Alexa Fluor 488-labeled phalloidin were purchased from ThermoFisher. Cytochalasin D and jasplakinolide were purchased from Sigma and reconstituted in DMSO and 100% ethanol, respectively.

### Plasmids and virus stocks.

A/WSN/1933 (H1N1) virus was generated by reverse genetics (78) using the 8 pPolI plasmids carrying different segments of IAV genome and the 4 pCAGGS plasmids that express the PA, PB1, PB2, and NP proteins. The titers of the stocks were determined using the plaque assay with MDCK cells. A/Wyoming/03/2003 (H3N2) [Wyoming (H3N2)], A/Panama/2007/1999 (H3N2) [Panama (H3N2)], and A/California/04/2009 (H1N1) [California (H1N1)] viruses were kind gifts from Arnold S. Monto (University of Michigan) and were received as low-passage-number stocks (less than 5 passages in MDCK cells) of virus isolated from clinical specimens. Virus infection was performed and monitored using the plaque assay and flow cytometry as described in Text S1 in the supplemental material.

10.1128/mBio.01916-18.1 TEXT S1 Supplemental methods: details of methods for virus infection and detection of infection are described. Download Text S1, DOCX file, 0.1 MB. Copyright © 2018 Bedi et al. 2018 Bedi et al. This content is distributed under the terms of the Creative Commons Attribution 4.0 International license.

### Measurement of vRNA levels.

Virus-containing cell culture supernatants were centrifuged at 3,000 rpm for 5 min in a microcentrifuge, filtered through a 0.45-μm filter, and subjected to ultracentrifugation at 30,800 rpm (AH650 swinging bucket rotor, ThermoFisher) for 90 min to prepare virus pellets. Virus and cell-associated vRNA was measured using a previously described protocol (79). Briefly, total RNA was extracted from virus pellets and cell lysates using TRIzol reagent (Ambion) according to the manufacturer’s protocol. cDNA was generated using random hexamer priming and the SuperScript III First-Strand Synthesis System (Invitrogen). Quantitative PCR was performed on a CFX96 Real Time PCR system (Bio-Rad) using Platinum SYBR Green pPCR SuperMix-UDG (ThermoFisher Scientific). Serial 10-fold dilutions of pPolI plasmids containing specific viral genes of WSN were used to generate a standard curve for quantification of cDNA copy number based on cycle threshold (*C_T_*) values. The primer sequences are shown in Text S1 in the supplemental material.

### Correlative fluorescence and scanning electron microscopy (CFSEM).

CFSEM experiments were performed as described before (80). Briefly, cells cultured on gridded coverslips (Bellco Biotechnology) were infected with WSN at MOI 0.1. Cells were fixed with 4% PFA in PBS at 20 hpi. After rinsing in PBS, quenching of PFA with PBS containing 0.1 M glycine (Sigma), and blocking with PBS containing 3% bovine serum albumin (BSA, Sigma), cells were immunostained with mouse anti-HA and fluorescently labeled secondary antibody. Cells were imaged using a Leica Inverted SP5X confocal microscope with a 40× PL APO objective and 10 to 20× scanning zoom. After fluorescence imaging, cells were fixed with PBS containing 2.5% glutaraldehyde (Electron Microscopy Sciences), stained with 1% OsO_4_, dehydrated in a series of ethanol washes, rinsed in hexamethyldisilazane (Electron Microscopy Sciences), and allowed to dry overnight. Coverslips were affixed to specimen mounts and sputter coated with gold for 90 s (Polaron). Cells were identified by their location on the gridded coverslip and imaged on an Amray 1910FEG scanning electron microscope at 5 to 10 kV. Fluorescence and SEM images were roughly brought into registration by scaling and rotating images in Adobe Illustrator, similarly to other correlative fluorescence/SEM studies (80). Landmarks used for registration included cell edges. Cell surface structures visible in SEM were manually classified as virus-like buds if they appeared spherical and near 100 nm in diameter. To identify HA clusters in fluorescence images unambiguously, we removed uniform nonpunctate HA signal from the images. To do this, we calculated a 20-pixel radius median filter and subtracted the median filtered image from the original using the *Image Calculator* function in ImageJ. The number of HA-positive puncta was measured in the background-subtracted fluorescence images using the *Analyze particle* function in ImageJ. Since MDM have substantial membrane folds on the cell surface, especially toward the center of the cell, we focused on areas toward the edge of the cells, which have a flatter topology, for quantification of efficiency of virus bud formation.

### *In situ* proximity ligation assay (PLA).

PLA was performed using the Duolink PLA fluorescence kit following the manufacturer’s instruction (Sigma). Cells fixed with 4% PFA (nonpermeabilized) were incubated with the following primary antibody combinations: goat anti-HA and mouse anti-M2 for PLA and rabbit anti-NA for identification of infected cells, mouse anti-HA and rabbit anti-NA for PLA and goat anti-HA for identification of infected cells, or goat anti-HA and mouse anti-TfR for PLA and rabbit anti-NA for identification of infected cells. Detection of PLA signals and identification of infected cells were performed using PLA probes specific to goat, mouse, or rabbit IgG and Alexa Fluor-488-labeled secondary antibody recognizing anti-NA or anti-HA, respectively. Cells were observed using a Leica Inverted SP5X Confocal Microscope System with a 63× objective. Z-stacks extending from the focal plane corresponding to the middle plane of the nucleus (identified by DAPI staining) to the bottom of cells were acquired for each cell, and the maximum intensity projection for each cell was constructed using ImageJ. The PLA signal in projection images was thresholded to eliminate weak and hazy background signal in the nucleus, and the number of PLA-positive spots was counted using the *Analyze particle* function in ImageJ.

### Actin fractionation assay.

Actin fractionation was performed as previously described (51). Briefly, dTHP1 cells were treated with 0.5% ethanol (vehicle) or 1 μM jasplakinolide for 4 h. Cells were incubated with cytoskeleton stabilization buffer (4 M glycerol, 25 mM PIPES, pH 6.9, 1 mM EGTA, 1 mM CaCl_2_) containing 0.1% Triton X-100 for 2 to 3 min. Cells were centrifuged for 5 min at 7,500 × *g* at 4°C. The pellet was resuspended in the cytoskeleton stabilization buffer. Supernatant (S) and pellet (P) fractions were run on a reducing and denaturing polyacrylamide gel and analyzed by immunoblotting.

### Statistical analysis.

Statistical analyses were performed using GraphPad Prism version 7. Two-tailed paired Student’s *t* test was used to calculate *P* values in Fig. 1 to 3 and Fig. S1 to S4. Two-tailed unpaired Student’s *t* test was performed in Fig. 4 to 7.
