# Supplementary material for: A Defect in Influenza A Virus Particle Assembly Specific to Primary Human Macrophages
Source: mBio. 2018 Oct 23;9(5):e01916-18. doi: 10.1128/mBio.01916-18 (PMC6199496; doi:10.1128/mBio.01916-18)
Supplement: TEXT S1 [file mbo005184122s1.docx]

**Supplementary Methods**

**Virus infection:** For virus infection, cells were first washed twice with MEM-BSA medium (1X MEM supplemented with 0.3% BSA (Sigma), 0.23% sodium bicarbonate (Gibco), 1X MEM amino acids (Gibco), and 1X MEM vitamins (Gibco)). The cells were then inoculated with diluted virus at MOI 0.01 or 0.1 for 1 hour at 37°C, washed twice with MEM-BSA, and cultured in MEM-BSA containing 0.2 μg/ml tosylsulfonyl phenylalanyl chloromethyl ketone (TPCK)-treated trypsin (Worthington).

**Infectious virus measurement by plaque assay:** Confluent monolayers of MDCK cells (cultured up to passage 40) plated in 24-well plates were washed twice with MEM-BSA and incubated with 100 μl of serial dilutions of virus supernatants for 1 hour at 37°C. Cells were washed once with MEM-BSA and layered with MEM-BSA containing 0.76 ug/ml TPCK-treated trypsin and 1% Seakem GTG agarose /Seaplaque (Lonza). After gelation of agarose, the plates were inverted and incubated at 37°C. After 48-72 hours, the agarose was removed, and the cells were stained with crystal violet (0.1% crystal violet in 20% methanol) for 10 minutes before counting plaques. The limit of detection of the plaque assay is 10 PFU/ml.

**Flow cytometry:** For viral protein expression analysis by flow cytometry, virus- or mock-infected cells were detached using 0.06% trypsin-EDTA in phosphate-buffered saline (PBS) and fixed with 4% paraformaldehyde (PFA, Electron Microscopy Sciences) in PBS for 20 minutes at room temperature. After fixation, cells were washed twice with 2% FBS in PBS (FACS buffer). For detecting viral protein expression on the cell surface, cells were subsequently incubated with primary antibodies for 45-60 minutes directly. For staining of intracellular proteins, cells were first permeabilized with 0.1% TritonX-100 in PBS for 5 minutes and then probed with primary antibodies. After washing once with the FACS buffer, cells were incubated with fluorescently labeled secondary antibodies (Invitrogen) for 20-30 minutes. Cells were washed twice with the FACS buffer and analyzed using the FACSCanto flow cytometer (BD Biosciences). Data were analyzed in FlowJo (Treestar), and the positive gates were set using the mock-infected controls.

**Primers for vRNA measurement**

The following primers were used for detection of vRNAs:

PB1 forward: 5’-TCAGAGAAAGAGACGAGTGAG-3’, PB1 reverse: 5’- AAACCCCCTTATTTGCATCC-3’

PB2 forward: 5’- GTTGGGAGAAGAGCAACAGC-3’, PB2 reverse: 5’-GATTCGCCCTATTGACGAAA-3’

PA forward: 5’- TGTGCAGCAATGGATGATTT-3’, PA reverse: 5’- TCTCCCATTTGTGTGGTTCA-3’

NP forward: 5’- GCGCCAAGCTAATAATGGTG-3’, NP reverse: 5’-GGAGTGCCAGATCATCATGT-3’

M forward: 5’-GACCRATCCTGTCACCTCTGAC-3’, M reverse: 5’- AGGGCATTYTGGACAAAKCGTCTA-3’

NS forward: 5’- CAGAATGGACCAGGCGATCA-3’, NS reverse: 5’-TAGAGTCTCCAGCCGGTCAA-3’

HA forward: 5’-TAACCTGCTCGAAGACAGAC-3’, HA reverse: 5’- AGAGCCATCCGGTGATGTTA-3’

NA forward: 5’- TTGGTCAGCAAGTGCATGTC-3’, NA reverse: 5’- ACAGCCACTGCTCCATCATC-3’.
